# P-753. Applying artificial intelligence to understand antibiotic resistance patterns of Staphylococcus aureus causing skin and soft tissue infections in children

**DOI:** 10.1093/ofid/ofaf695.964

**Published:** 2026-01-11

**Authors:** Lilly Immergluck, Abdolreza Mozaddegh, Samuel Owusu, Chaohua Li, Traci Leong, Xiting Lin, Declan Quinn, Peter T Baltrus, Robert C Jerris

**Affiliations:** University of Chicago, Chicago, IL; Northeastern University, Oakland, California; Morehouse School of Medicine, Atlanta, Georgia; Morehouse School of Medicine, Atlanta, Georgia; Emory University, atlanta, Georgia; Morehouse School of Medicine, Atlanta, Georgia; University of Chicago, Chicago, IL; Morehouse School of Medicine, Atlanta, Georgia; Emory University School of Medicine/Children's Healthcare of Atlanta, Atlanta, Georgia

## Abstract

**Background:**

Antimicrobial resistance (AMR) continues to grow worldwide. For *Staphylococcus aureus* (*S. aureus*), antibiotic resistant phenotypes have expanded in community settings, especially for skin/soft tissue infections (SSTIs). Resistance to clindamycin and other non-beta lactam antibiotics leads to multidrug resistance (MDR)*S. aureus.*Association mining is an unsupervised machine learning algorithm that examines higher-order relationships between resistant antibiotics. It can quantify prevalence (support) and strength of association (lift). We apply artificial intelligence (AI) using AM to understand MDR *S. aureus* patterns in children with SSTIs over time.

**Methods:**

Data on children with SSTI seen in a large pediatric healthcare system in Atlanta, GA, U.S.A. were obtained retrospectively (2002-2019). Using AI, we applied association rule mining to look for antibiotic resistant patterns meeting the criteria: *S. aureus* SSTI from patients < 19 Y living in Atlanta. Association rule mining applied to identify antibiotic resistant phenotypes most frequent/clinically relevant (using *Apriori* algorithm), based on arules package in R. The following antibiotic classes were included in the analyses:clindamycin, erythromycin, gentamicin, linezolid, oxacillin, rifampin, trimethoprim-sulfamethoxazole, tetracycline, and vancomycin. Interestingness measures, including support (prevalence of resistance patterns) and lift (strength of associations between resistances) were employed to extract frequent and reliable patterns and then stratified by year and methicillin susceptibility. Analyses conducted in R.

**Results:**

Preliminary analyses of subset (2002-2016) of data shows 40 distinct MDR *S. aureus* phenotypes from 8,171 children with SSTI. Clindamycin resistance was found in 19/40 (48%) phenotypes, 27/40 (68%) phenotypes had >3 resistant antibiotic classes, 11/40 (28%) occurred only in later years, and 4/40 (10%) had persistent phenotype through entire study period.

**Conclusion:**

Trends of MDR*S. aureus* are clustered around specific phenotypes. Association mining of *S. aureus* phenotypes across time can benefit antimicrobial stewardship programs by demonstrating which resistant patterns are circulating in the community setting.

**Disclosures:**

Lilly Immergluck, MD, MS, American Academy of Pediatrics: Board Member|Department of Energy: Grant/Research Support|moderna: Grant/Research Support|NIH: Grant/Research Support|Pfizer: Grant/Research Support|Sanofi: Grant/Research Support

